# Geospatial forest fire risk assessment and zoning by integrating MaxEnt in Gorkha District, Nepal

**DOI:** 10.1016/j.heliyon.2024.e31305

**Published:** 2024-05-18

**Authors:** Gayatri Paudel, Kabita Pandey, Puspa Lamsal, Anita Bhattarai, Aayush Bhattarai, Shankar Tripathi

**Affiliations:** a Institute of Forestry, Tribhuvan University (TU), Nepal; b Faculty of Forestry, Agriculture and Forestry University (AFU), Nepal

**Keywords:** Forest fire, Risk assessment, MaxEnt model, Environment variables, Zoning

## Abstract

Forest fires are an imminent danger to natural forest ecosystems, and carrying out zoning studies and forest fire risk assessments are of great practical significance in steering fire prevention, minimizing fire incidents, and limiting the environmental consequences of fire. Using the Gorkha district of Nepal as a case study, this study used remotely sensed high-temperature fire data as the forest fire sample. Nine parameters related to topography, climatic conditions, vegetation, and human intervention were used as environmental variables affecting fire occurrence. Next, a MaxEnt forest fire risk assessment model was generated with GIS and R, which analysed the contribution, significance, and responses of environmental variables to the forest fire in Gorkha District. The findings demonstrate that (1) following a test of sample locations for forest fires, the MaxEnt model has excellent relevance and practicality when applied to fire risk assessment; (2) Out of 2747 fire points in the forest, only 110 Spatio-temporally independent fire points were used for the model building having high and normal confidence level. Regarding Area Under Curve (AUC) values, the training data yielded results of 0.875, while the test data produced acceptable results of 0.861 with a standard deviation of 0.0322; (3) the importance of climatic and Land Use Land Cover (LULC) variables to forest fire are 56.2 % and 32.9 %, respectively, and their contribution to forest fire are 32 % and 47.6 %, respectively. (4) There are numerous and intricate ways that environmental factors influence forest fires. The forest fire response curves to the nine chosen environmental variables are complex and nonlinear rather than linear; Maximum temperature of the warmest month (bio_5), Isothermality (bio_3), Precipitation of Driest Quarter (bio_17) and mean Diurnal Range (bio_2) bear a nonlinear positive link with the possibility of forest fires. In contrast, elevation, slope, temperature seasonality (bio_4), distance from the settlement, and LULC have a favorable stimulating response to the possibility of forest fires within an appropriate interval. (5) In Gorkha, there are geographical differences in the risk of forest fires. Only 12.83 % of the whole area is made up of areas at significantly high risk or above, compared to 87.17 % for high-risk and below.

## 1 Introduction

Globally, in a thousand of forest fires, over a million hectares of woodland are burned every year severely disturbing nature's equilibrium, and endangering people's lives and socioeconomic progress [1]. Recently, forest fire risks have been rising, and this might contribute to significant disruptions to the ways several ecosystems function [2,3]. The Australian fires [4] and the Amazon fires [5] occurred as a result of inadequate local forest management techniques and changing climates, causing an immense decline in biological diversity. Forest fire trend is increasing around the world [6].

In Nepal, a key factor contributing to the destruction of forests is rampant forest fires. Due to human activity, the majority of forest fires—of which 89 % happen in March, April, and May—occur during the dry season [7]. Forest fire incidents have been increasing annually in Nepal [8]. The record of fires is very high during the dry season and this has severely affected the natural vegetation as well as public property [9]. The sporadic fire grievously damages vegetation, threatens biodiversity, declines ecosystem services, prohibits regeneration and seedlings development, destroys Non-Timber Forest Products (NTFPs), encourages invasive species, and causes damage to human life and property [10,11]. Forest fires in Nepal have been growing yearly [8]. There are an abnormally high number of fires during the dry season, which severely damages both public land and natural vegetation [9]. Bhujel et al. (2016) [8] report that between 2000 and 2016, Nepal experienced 35,374 fire incidents and a total scorched area of 1,723,920 ha. Furthermore, Bhujel et al. (2016) [8] estimate that the value of the forest resources destroyed as a result of the fire is US$107,798. The assessment also includes 11 fatalities and 100 injuries. One of the main factors causing Nepal's forest ecosystem disruption and biodiversity loss is human-caused fire [12]. During peak fire season in the month of April and May, many forest fires are seen in different places. From Gorkha district, the rampant forest fire news get published annually in various national newspapers [13].

Parajuli et al. (2020) [14] developed forest fire risk models in Terai Arc Landscape (TAL) and Chitwan Annapurna Landscape (CHAL), two of Nepal's most significant landscapes, using remote sensing, GIS technology, and statistical methods. As an early warning measure, fire-risk maps are extensively created in several nations at coarse resolution employing wildfire fuel models or vegetation maps [15,16]. According to Jaiswal et al. (2020) and Erten et al. (2004) [17,18], a fire risk zone is an area where there is a chance of having fires that could spread to nearby areas. To reduce the potential effects of forest fires, precise threat zone mapping is crucial [17].

Zoning studies offer a crucial scientific foundation for developing forest fire risk defense systems, deploying firefighting staff, and directing preventive efforts as part of the evaluation of forest fire risk. Global organizations and academia are paying more and more consideration to fire risk assessment [19,20]. The reliability of forest fire danger zones depends on the creation of quantitative evaluation models. Of these, the maximum entropy (MaxEnt) model is a more sophisticated one that has been effectively used in recent years in forest fire risk zoning studies conducted globally [21]. MaxEnt chooses the optimal distribution by taking into account the known sample data at a single moment in time and the environmental variables. The optimal distribution is the one that has the highest entropy for each environmental variable. The model is built with the intention of anticipating the sample events' spatial distribution [21,22]. Due to the fact that it is easily used, has little input data prerequisites, and is readily created, the MaxEnt model has been effectively used in forest fire risk zoning research [23]. Many forest fire prediction mappings have made use of MaxEnt. Research on the prediction of forest fires shows that MaxEnt outperformed other machine-learning methods [[24], [25], [26], [27]]. Based on historical fire data and environmental variables in the Daxinganling Mountains from 2005 to 2010, the MaxEnt model was used to predict the possibility of lightning fire occurrence in a 1 km grid. It was discovered that the MaxEnt model can be used to predict lightning 36 fire occurrence in the Daxinganling Mountains. This model can be used by forest managers to assess the daily fire hazard in different locations [28].

Satellite data and models are widely utilised for risk mapping, danger predicting, and fire tracking. Geospatial models have been used to map fire risk indexes in different parts of the globe [17,23,29,30]. Investigating and making an effort to forecast the locations and times when fires are most likely to occur is also crucial. This knowledge is essential for figuring out what causes forest fires, creating strategies to lessen their frequency, controlling and managing sources of combustion, and figuring out high-risk areas [31,32]. Because of this, it is necessary to construct an integrated forest fire risk model and to use zonation that takes into account biophysical and human aspects. Furthermore, Matin et al. (2017) [7] stated in his study that forest managers at the national and regional levels will be able to distribute resources across districts according to relative hazards with the use of district-level risk maps. Ministry of Forest and Environment (MoFE) and the International Centre for Integrated Mountain Development (ICIMOD) (2019) also released a fire danger map of Nepal, with Gorkha included as one of the high-risk districts [33]. Despite the need for district-level risk maps revealed by Ref. [7], the study is expected to influence the local level because there is a gap in the research in the district-level study of fire pattern and fire risk mapping. Kunwar and Khaling (2006) [34] mentioned in the study that Local communities play a significant role in preventing and suppressing harmful fires because they have clear understanding of local conditions and circumstances important for successful fire management. As a result, this research was conducted to evaluate the danger of forest fires and to zone the risk regions in Nepal's Gorkha district.

## 2 Materials and methods

### 2.1 Study area

**Fig. 1:**
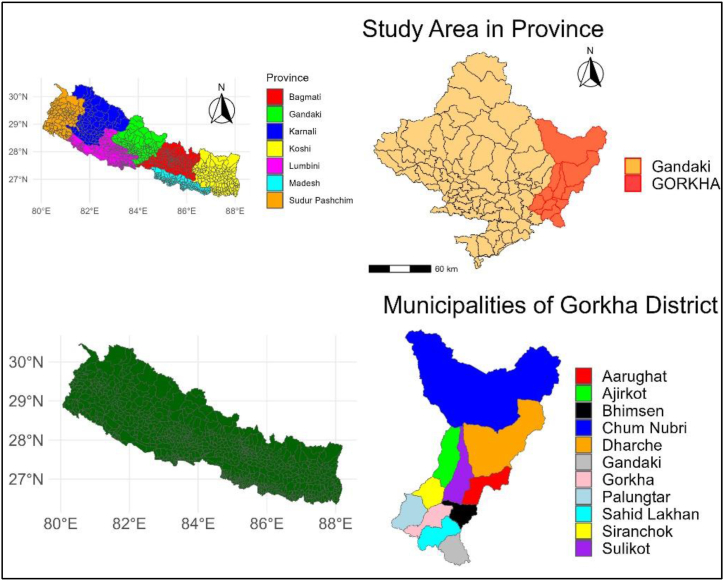
Study area map.

The study was conducted in the Gorkha District of Nepal. The fourth-largest district in Nepal, out of 77 total, is found in Gandaki Province (Fig. 1). Two urban and nine rural municipalities make up the district's eleven municipalities. Gorkha district lies between the latitude of 28° 28′ 35.0220″ North and the longitude of 84° 41′ 23.1036″ East. It extends over an area of 3610.70 square kilometers. The elevation ranges from 330 m (Trishuli River bank) to 8,156 m (Mt. Manaslu) above sea level. The climatic zone varies from the lower tropical zone in the South to the Trans-Himalayan zone in the North. The average yearly precipitation is 254.9 mm [35]. The annual highest and lowest temperature varies from 19.4 °C to 10.1 °C [35]. The vegetation types vary from tropical Salforest, temperate Pine, and Rhododendron Forest to rocky pastureland. Leopard, Ghoral, Badel, Pangolin, etc. are the animal species found in the forest [35].

### 2.2 Materials

#### 2.2.1 Environmental variables

Environmental variables include bioclimatic, topographical, and anthropogenic variables.

#### 2.2.2 Fire incidence data (https://firms.modaps.eosdis.nasa.gov)

To conduct this study, incidence data of the forest fires for the period of 2012–2021 of Visual Infrared Imaging Radiometer Suite onboarded on Suomi National Polar-Orbiting Partnership Spacecraft (VIIRS-SNPP) was downloaded from Fire Information for Resource Management System of National Aeronautical Space Administration.

## 3 Methodology

**Fig. 2:**
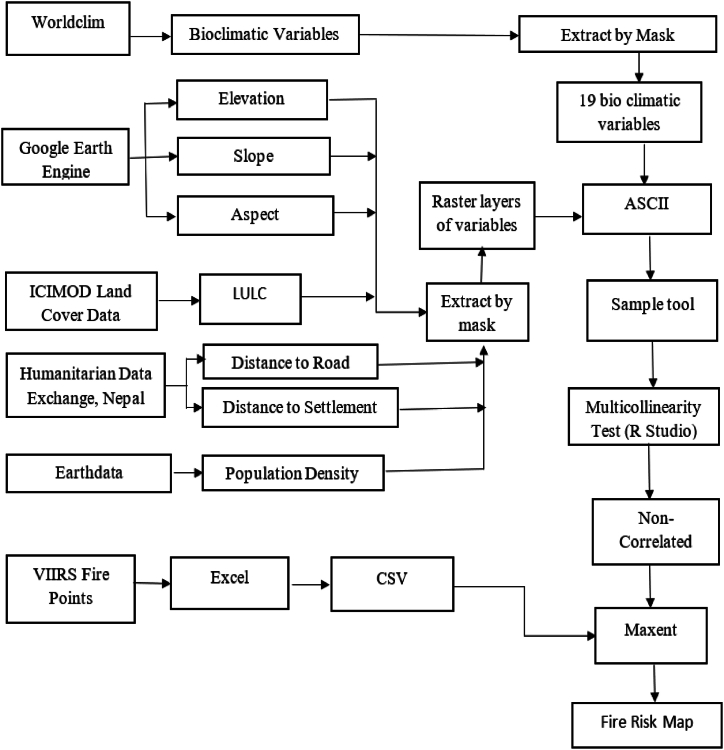
Methodological flowchart.

The methodology involves four main steps 1) Fire points’ preparation for modelling, 2) altering environmental layers to the same extent (geographic projection and cell size) using ArcGIS, 3)correlating environmental variables and removing multicollinearity, and 4) model building as presented in (Fig. 2).

### 3.1 Fire points' preparation for modelling

The preparation of fire occurrence data for Modelling includes following steps.

#### 3.1.1 Filtering fire points

Out of 3630 fire incidences, only 110 filtered fire incidences were used in modelling. For the present study, the VIIRS-SNPP fire points were filtered first using high (h) and normal(n) confidence under the Confidence of attribute fields, then filtered using acquisition date with fire season of Gorkha and then the fire point was filtered using LULC to remove the fire points falling outside the forest areas.

#### 3.1.2 Spatial auto-correlation test of fire points

After filtering, a total of 2747 fire incidences were found in the forest-covered areas. However, when it runs spatial autocorrelation moran's I indices there exist correlation within the fire points. Buffer of 1 km was created and the points were deleted for overlapping buffer. Then all the fire point of buffer distance was run for the spatial autocorrelation. The result of spatial autocorrelation of 1 km buffer distance with 110 fire point was finalized to use in this study.

#### 3.1.3 Producing a comma-separated value (.csv)

The fire points were saved as comma-separated value files using MS Excel. Only three column headings titled: Fire, Longitude, and Latitude were kept in the .csv file.

### 3.2 Modifying environmental layers

All the environmental layers should be in raster format and have the exact cell size and projection system (e.g., geographic or UTM) to execute a forest fire model. In this study all the layers were re-sampled to 30 m pixel size and UTM WGS 1984 Zone 45 projection. Road, Settlement, and LULC were the Land cover predictors on which Road and Settlement were converted from discrete vector format to continuous raster using the Euclidian distance tool and LULC was converted from continuous vector to continuous raster format. Topographic Predictors such as Slope, Aspect, and Elevation were generated from SRTM DEM using relevant surface tools of spatial analyst. For maintaining consistency, layers were projected to UTM Zone 45, pixel size resampled to 30 m, as well as masked by the boundary area of the study area using the extract by mask tool. All the layers were converted to ASCII file format which is friendly to MaxEnt Software.

### 3.3 Correlation test of environmental variables

The ENM Tools version 1.3 [36] was used to conduct a correlation test of various environmental layers to examine multi-collinearity between predictor variables. Statistical research revealed that certain of the sample locations' bioclimatic characteristics have a very strong association. To provide clarity among bioclimatic variables that had shown commonalities, Pearson Correlation Coefficients were applied. The correlation coefficient might be negative or positive. A negative correlation value implies that the value of one variable declines as the value of the other variable rises, whereas a positive correlation value shows that the value of one variable rises as the value of the other variable rises. A correlation value of 0.0 indicates that the variables are unrelated: a positive increase in one variable is unrelated to a positive or negative change in the other. We can assess whether or not two separate variables will have a distinct spatial influence on the distribution of the species using the r value. Variables with r > 0.7, i.e. variables correlating more than 70 %, were no longer considered in analysis and model construction as the high correlation [37] between independent variables impacts on model accuracy with multi-collinearity (Table 2).

**Table 1:** Variables used for multi-collinearity test.

Environmental Variables	Code	Source
Climatic Variables		
Annual Mean Temperature	*bio1*	WorldClim
Mean Diurnal Range (Mean of monthly (max temp – min temp)	*bio2*	
Isothermality (P2/P7) *(100)	*bio3*	
Temperature Seasonality (standard deviation*100)	*bio4*	
Max Temperature of Warmest Month	*bio5*	
Min Temperature of Coldest Month	*bio6*	
Temperature Annual Range (P5–P6)	*bio7*	
Mean Temperature of Wettest Quarter	*bio8*	
Mean Temperature of Driest Quarter	*bio9*	
Mean Temperature of Warmest Quarter	*bio10*	
Mean Temperature of Coldest Quarter	*bio11*	
Annual Precipitation	*bio12*	
Precipitation of Wettest Month	*bio13*	
Precipitation of Driest Month	*bio14*	
Precipitation of Seasonality (Coefficient of Variation)	*bio15*	
Precipitation of Wettest Quarter	*bio16*	
Precipitation of Driest Quarter	*bio17*	
Precipitation of Warmest Quarter	*bio18*	
Precipitation of Coldest Quarter	*bio19*	
**Topographic Variables**		
Elevation (m)	*elevation*	SRTMDEM
Slope (°)	*slope*	SRTMDEM
Aspect (°)	*aspect*	
**Land Use Land Cover**	*LULC*	ICIMOD
**Anthropogenic Variables**		
Distance to Road	*dr*	Humanitarian Data Exchange, Nepal
Distance to Settlement	*ds*	
Population Density	*pd*	Earth data

**Table 2:**
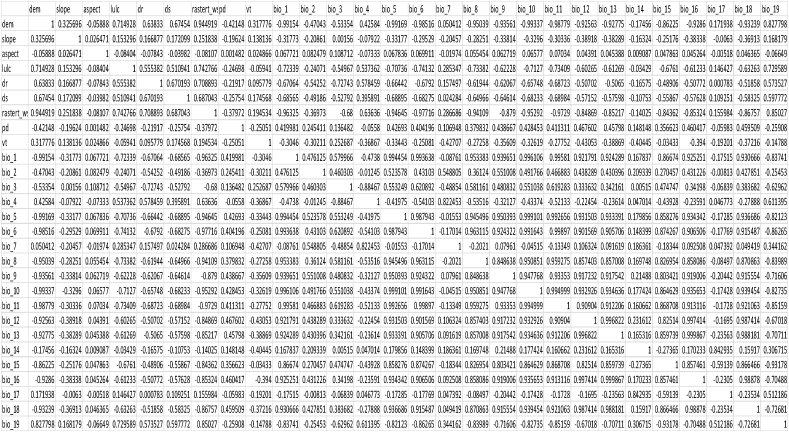
Correlation test result.

Fig. 2 shows the extended process we applied during this research with different variable and different stages.

**Fig. 3:**
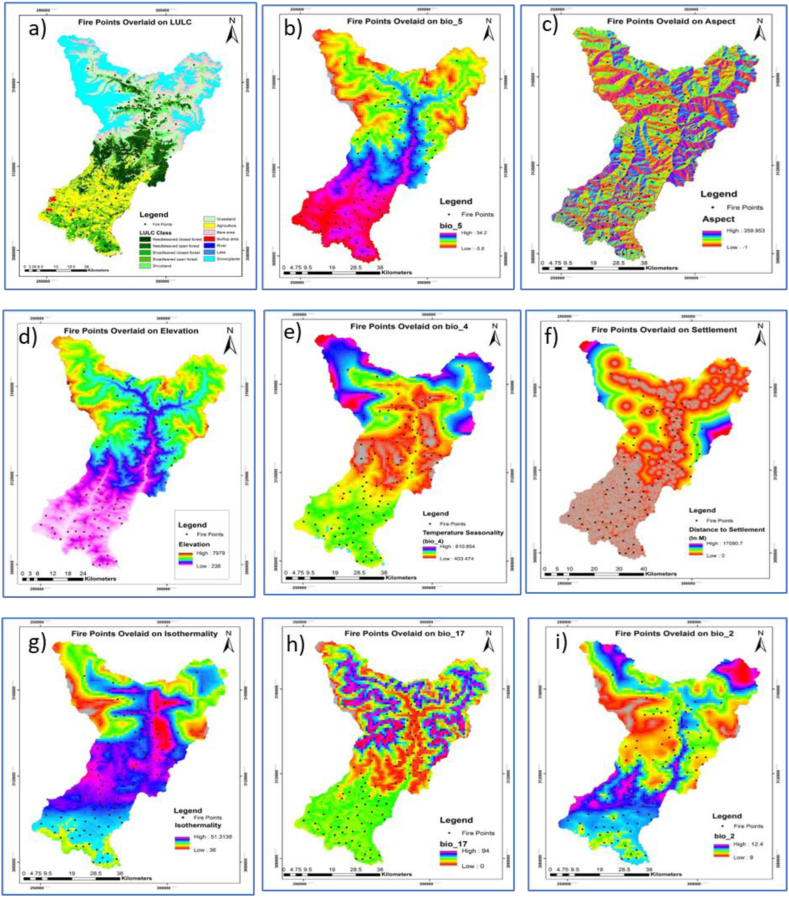
The variables influencing forest fire, that were used are as follows: a) LULC type; b) Maximum Temperature of the Warmest month (bio_5); c) aspect; d) elevation (dem); e) Temperature Seasonality (bio_4); f) Settlement (ds); g) Iso-thermality (bio_3); (h) Precipitation of the Driest Quarter (bio_17); i) Mean Diurnal Range (bio_2).

A total of nine variables were used in wildfire classification and the assessment of forest fire risk i.e. LULC (Fig. 3a), Bio_5 (Maximum Temperature of Warmest Month) range of the Gorkha varies from −5 °C to 34.2 °C as (Fig. 3b), Aspect (Fig. 3c), Elevation (Fig. 3d), Bio_4 (Temperature Seasonality) (Fig. 3e), settlement (Fig. 3f), Isothermality (Fig. 3g), Bio_17 (Precipitation of Driest Quarter) of the Gorkha ranges from 0 mm to 94 mm (Fig. 3h) and Bio_2 (Mean Diurnal Range) (Fig. 3i). Fire points were overlaid with this variable for better visual understanding (Fig. 3).

### 3.4 MaxEnt modelling

All these nine variables and fire points were imported modelling of fire risk was performed using MaxEnt. AUC was observed to assess the model's accuracy. Fair or good AUC values typically range between 0.7 and 0.8; with values above 0.8 being considered very good, and those values surpassing 0.899 are considered excellent; whereas AUC values below 0.5 are considered unfavourable [38]. Potential forest fire risk zones were identified with the produced map. The MaxEnt Jackknife test was used to evaluate the relative strengths of predictor variables by identifying the variable with the highest gain in model performance when used in isolation. The gain is calculated as the log of the number of grid cells minus the log loss, expressing the average probability of the point localities.

## 4 Results and discussion

### 4.1 MaxEnt forest fire model accuracy

**Fig. 4:**
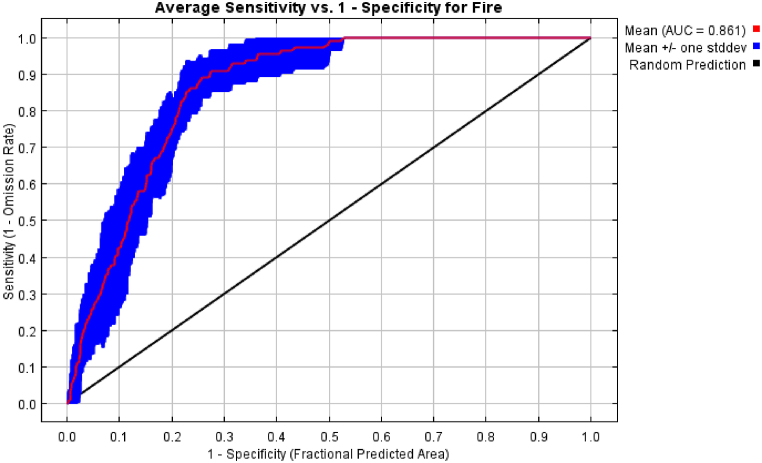
Auc curve.

A total of 3630 VIIRS-SNPP fire points were recorded in the Gorkha during the last 10 years from 2012 to 2021. Among them, 2747 fire incidence were seen in the forest areas. Out of 2747 fire points in forest, only 110 spatio-temporally independent fire points were used for model building having high and normal confidence levels. Model Accuracy was performed with the AUC value and obtained AUC 0.861 and with a standard deviation of 0.0322 which indicates that the performed modelling was with high accuracy in comparison to random perdition i.e. red curve (AUC) is far from the black curve (random prediction) (Fig. 4) which falls under the very good category of model accuracy [38].

### 4.2 Risk zone area map

Out of the total geographical area of 361,000 ha, the model predicted that 46598.49 ha is under very high-risk areas, 51971.31 ha under high-risk areas, 59720.85 ha under moderate risks areas, and 205269.3 ha under low risks areas as shown in Table 3.

**Table 3:** Areas under different fire risk zone classes.

Fire Risk	Area in Ha	Percentage (%)
Low	205269.3	56.46
Moderate	59720.85	16.42
High	51971.31	14.29
Very High	46598.49	12.83

**Fig. 5:**
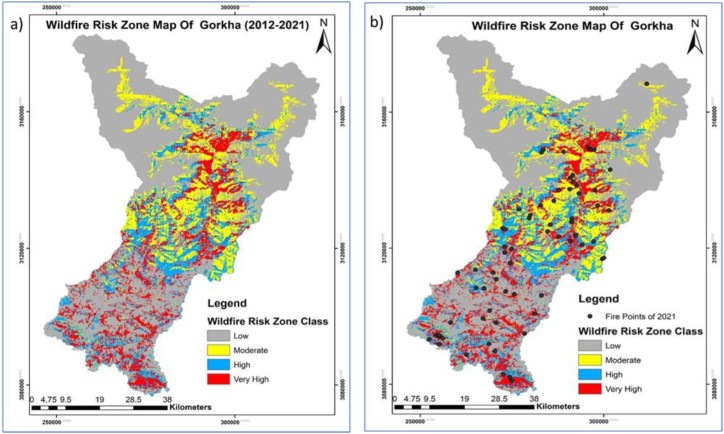
Forest Fire: a) Risk zoning, and b) Fire observed in 2021.

The spatial pattern of forest fire risk was obtained with the help of fire incidents between 2012 and 2021 and nine bioclimatic variables which provide more detailed insight into potential forest fire risk zones (Fig. 5a and b). The areas indicated by red and blue were under very high and high risk whereas areas with yellow and grey color were on less risk with moderate and low risk (Fig. 5a and b), whereas black dots were the recent fire of 2021 (Fig. 5b).

### 4.3 Predictor environmental variables performance

Estimates of the relative contributions of the environmental variables to the MaxEnt model are provided in the following table. The environmental variables with the highest percentage contribution in modelling forest fire risk zones were LULC (47.6 %), Maximum Temperature of the Warmest Month (bio_5) (25.9 %), Elevation (dem) (10.5 %) respectively (Table 4).

**Table 4:** Percent Contribution and Permutation importance of predictor variables.

Code	Variables	Percent Contribution	Permutation Importance
LULC	Land Use Land Cover	47.6	32.9
bio_5	Max Temperature of Warmest Month	25.9	47.4
Dem	Elevation	10.5	3.7
Ds	Distance to Settlement	4.0	3.0
aspect	Aspect	3.9	3.9
bio_4	Temperature Seasonality (standard deviation*100)	3.0	4.4
bio_3	Isothermality (P2/P7) *(100)	2.3	0.2
bio_2	Mean Diurnal Range (Mean of monthly (max temp – min temp))	0.6	1.7
bio_17	Precipitation of Driest Quarter	0.2	2.5

### 4.4 Relationships between environmental variables with forest fire

**Fig. 6:**
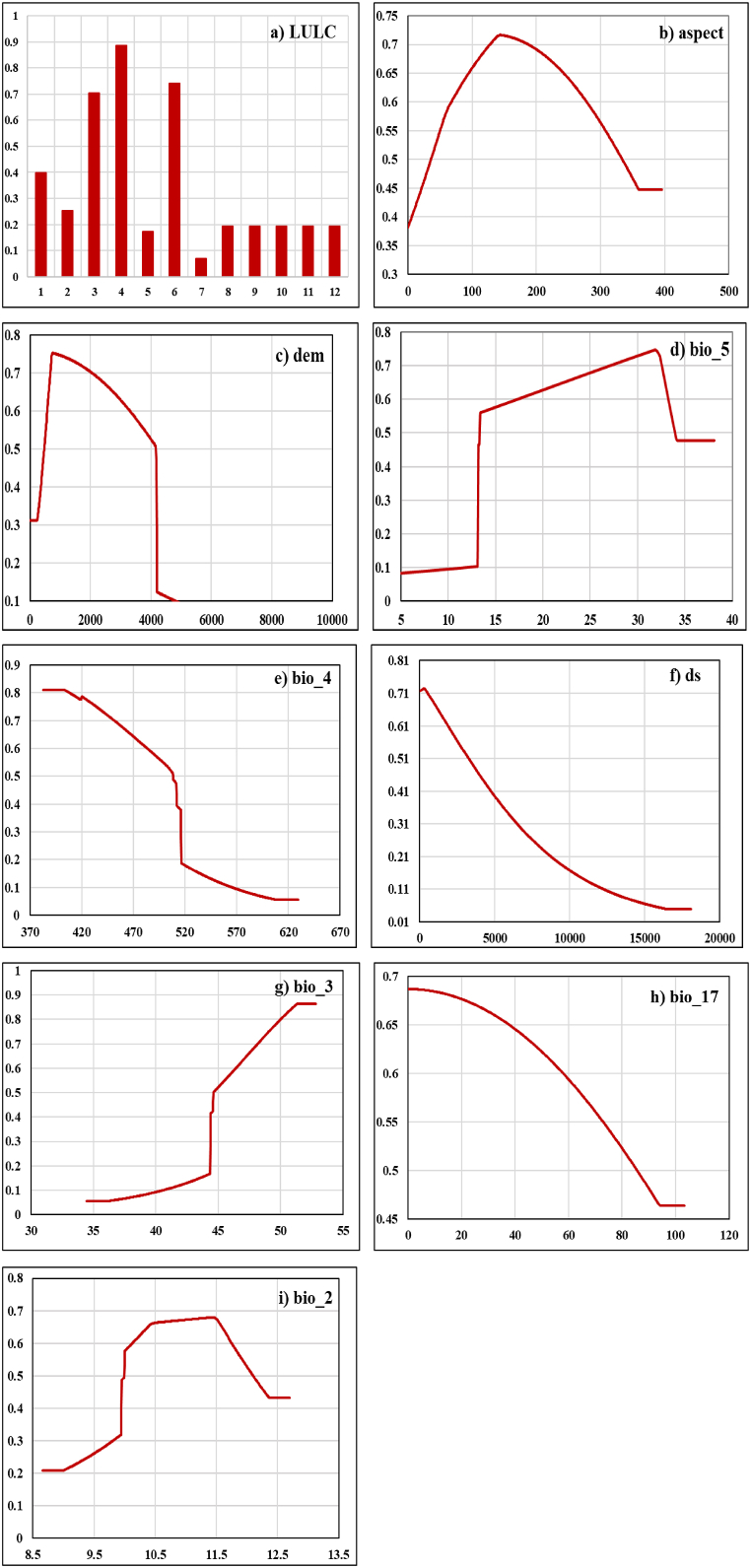
Response curves of environmental variables for forest fire with y-axis response and x-axis represent respective variable variances: [a] LULC, [b] aspect, [c] DEM, [d] bio_5 (Maximum Temperature of Warmest Month), [e] bio 4 (Temperature Seasonality, [f] ds (Distance to Settlement), [g] bio 3 (Isothermality), [h] bio 17 (Precipitation of the Driest Quarter), [i] bio 2 (Mean Diurnal Range).

The response curve generated from the MaxEnt model shows that LULC contribution in modelling of wildfire risk zone for the Gorkha was found to be the highest and most significant with 47.6 % contribution. Information about the main forest types and other land use categories in Gorkha was included in the LULC map. Based on the present study, it was found that the probability of Fire occurrence is higher at Class Code 4 which is the Broad-Leaved open forest followed by Class Code 6 and 3 which is Grassland and Broad Leaved closed forest respectively, which is shown in Table 5 and Fig. 6a. Human land use practices may serve as sources of combustion for regions that are susceptible to forest fires, and hence, the LULC predictor variable contributes the highest percent contribution (47.6) compared to other variables.

**Table 5:** Class code and class description for LULC.

Code	Class	Code	Class
1	Needle leaved closed forest	7	Snow/Glacier
2	Needle leaved open forest	8	Bare area
3	Broad leaved closed forest	9	Build up Area
4	Broad leaved open forest	10	Agriculture
5	Wetland	11	Shrubland
6	Grassland	12	Other

It is found that the aspect has a minor contribution with 3.9 % (Table 4) having the probability of the highest occurrence of fire found around to be 100 to 250, that is from the east to southwest aspect (Fig. 6b). In the study, it was found that elevation has a contribution of 10.5 % (Table 4). The probability of the highest occurrence of Fire was found to be in 500 m–4000 m elevation which is characterized by the presence of Broad-leaved to Needle-Leaved Forest and beyond which increasing elevation decreases the chance of wildfire as shown in Fig. 6c.

Max Temperature of the Warmest Month has a contribution of 25.9 % (Table 4). The response curve of Fire with Maximum Temperature of Warmest Month shows high forest fire between ranges of 15–35 °C as shown in Fig. 6d.

The response curve generated from the MaxEnt model shows that temperature seasonality (Bio 4) contribution in the modelling of forest fire-prone areas for Gorkha was found to be 3 % (Table 4) and fire risk was found high around 370 to 500 temperature seasonality after that fire risk decreased (Fig. 6e). Isothermality has a negligible contribution to the fire with 2.3 % (Table 3) and risk increased while isothermality increased (Fig. 6g).

The MaxEnt model's response curve demonstrates that the contribution percent and permutation importance of (bio_2) Mean Diurnal Range (Mean of monthly (max temp – min temp) to the modelling of Gorkha's forest fire-prone area was found to be 10 and 12 respectively (Fig. 6i).

The response of the probability of forest fire to distance from settlement is generally consistent with popular beliefs, i.e., the nearer the settlement, the greater the chance of forest fire occurrence. Specifically, the probability of forest fire gradually decreases or increases when the settlement distance is higher or lower than 2 km, respectively. However, it remains unchanged when the distance reached is 17 km or higher (Fig. 6f). The contribution of distance to settlement is 6 % (Table 4). This response pattern may be related to natural or man-made fire sources (Fig. 6f). When the distance to settlement is more than 15 km, human activities disturb fewer areas. In this case, wildfires are more likely to be caused by natural conditions, such as lightning fires, the spontaneous combustion of peaks, and sparks caused by rolling rocks. In contrast, with decreasing distance from settlement, human activities are more widespread, intensifying disturbance, so that the possibility of human-caused fire increases dramatically, thus increasing the likelihood of forest fire.

The Precipitation of the Driest Quarter (bio_17) is approximating the total precipitation in mm that prevails during the driest quarter (March to May). The percent contribution of Precipitation of Driest Quarter is 0.2 % (Table 4) which was found to be less significant. The results are interpreted from the response curve as shown in Fig. 6h. There is negative relationship of fire with Bio17 from 0 mm to 94 mm. Increased precipitation in the Driest Quarter can minimize the fire risk zone in the Gorkha.

### 4.5 Response curve of variables used

.

### 4.6 Model analysis of jackknife test

**Fig. 7:**
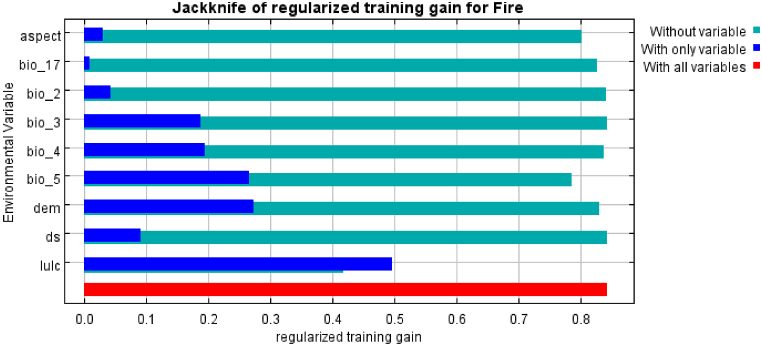
Jackknife of regularized training gain of Fire.

LULC is the environmental variable that yields the biggest benefit when employed alone, and as such, it seems to have the most valuable information on its own (Fig. 7). LULC is the environmental variable that, when removed, reduces the gain the most; as a result, it seems to contain the most information not found in the other variables. The displayed values are averages over multiple runs.

**Fig. 8:**
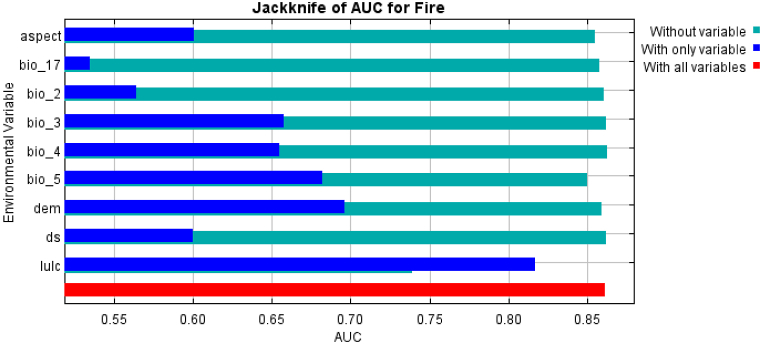
Jackknife of AUC for fire.

Lastly, it was performed the same jackknife test, using AUC on test data (Fig. 8). The figure reflects the impact of each variable on the entire model and reflects the function and signification of each variable in more detail. Light blue indicates the impact on the model if this variable is not included, and dark blue indicates the independent contribution of this variable to the model.

## 5 Discussion

Response curve generated from the MaxEnt model shows that LULC contribution in modelling of forest fire prone area was found to be the highest and significant contribution [39] and same variable has contributed highly to forest fire in the study area (Table 4, Fig. 5, Fig. 8). Dorji (2014), Saxena and Srivastava (2007) [40], [41] conducted research and developed a risk model by integrating biophysical characters, microclimate, and human factors similarly as this study (Table 1). This research showed that temperature plays a positive role in promoting forest fire; the probability of forest fire increases with increasing temperature (Fig. 6d and g). The record of fires is very high during the dry season and this has severely affected the natural vegetation as well as public property [9] as increasing temperature contributes in forest fire occurrence (Fig. 6, Fig. 7, Fig. 8). Anthropogenic activities also has great contribution to forest fires, the nearer the settlement, the greater the chance of forest fire occurrence [42]. The influence of human activities, such as land use change and urbanization, on forest fire occurrence and severity, highlights the need for integrated approaches to fire risk assessment [28]. Environmental variables have a complicated and varying impact mechanism on forest fires (Fig. 6, Fig. 7, Fig. 8; [43]). A complex nonlinear relationship rather than a linear response curve characterizes the forest fire reaction to all environmental variables (Fig. 6, Fig. 7). Table 3 illustrates that the proportion of locations at and above the very high-risk level for forest fires is rather low.

For assessing the danger of forest fires, a plethora of models are available, with binary logistics regression (BLR) being the most used model [43]. This study employed the fire spots and environmental elements as input for fire risk assessment in order to further investigate the rationale of employing the MaxEnt model. After testing forest fire sample sites, Yang et al. (2021) [43]demonstrated that the maxEnt model has excellent application and practicality when used to assess the danger of forest fires, as this study also has shown similar results with satisfactory accuracy (Fig. 7, Fig. 8). Similarly, Alcasena et al. (2021) [44] explored the effectiveness of different modelling techniques, including MaxEnt, for predicting wildfire occurrence and identifying high-risk areas, emphasizing the importance of considering both environmental and anthropogenic factors. Krawchuk et al. (2009) [45] conducted research that was also focused on understanding the complex interactions between climate, vegetation, and fire regimes, emphasizing the importance of incorporating these factors into fire risk models.

## 6 Conclusion

Forest fire risk assessment and zoning can be accomplished in an instinctive, comprehensive, and considerably precise technical manner by integrating the MaxEnt model with GIS to create a model employing verified forest fire spots and environmental variables. In order to perform a district-level forest fire risk assessment and zoning analysis, a MaxEnt forest fire risk evaluation model was built in this research with GIS technical assistance utilizing a 2012–2021 dataset of forest fire hotspots and nine environmental variables in Gorkha district, Nepal. Just 110 fire points that are spatially and temporally independent and have high and normal confidence levels were used for the model development out of a total of 2747 fire points in the forest. AUC values of 0.875 were obtained for the training data, while 0.861 with a standard deviation of 0.0322 were found to be appropriate outcomes for the test data. For forest fire, the importance of environmental variables in descending order includes climatic conditions, vegetation, topography, and human activities, while the contribution of environmental variables in descending order includes vegetation, climatic conditions, topography, and human activities. There are numerous and intricate ways that environmental factors influence forest fires. The forest fire response curves to the nine environmental factors that were chosen are complex and nonlinear; Maximum temperature of the warmest month (bio_5), aspect, Isothermality (bio_3), Precipitation of driest quarter (bio_17), and Mean diurnal range (bio_2) all show a nonlinear positive association with the probability of forest fires. On the other hand, the likelihood of a forest fire is positively promoted by elevation, slope, Temperature seasonality (bio_4), distance from settlement, and LULC when they are within an appropriate interval. It is clear that Gorkha's forest fire risk varies geographically. 87.17 % of the whole area was made up of the places with low to high risk, while only 12.83 % was made up of the areas with extremely high risk and above. According to the geographical distribution pattern, Chum Nubri Gaunpalika's Chumling, Dumje, and Chumjet areas are home to the majority of the extremely high-risk zones. Along with Gorkha Municipality, the areas of notably high risk are centered in Gandaki and Sahid Lakhan Gaupalika. The majority is found in the high- and moderate-risk zones, although it is unclear how the clustering is distributed geographically. The Chum Nubri Rural Municipality's Manaslu Conservations region, as well as the Gorkha district's Ajirikot, Siranchok, and Aarughat rural municipalities, are the locations with the highest concentration of low-risk zones.

## Funding statement

There was no funding source for this research.

## Data availability statement

Data will be made available on request.

## CRediT authorship contribution statement

**Gayatri Paudel:** Writing – original draft, Methodology, Formal analysis, Data curation, Conceptualization. **Kabita Pandey:** Methodology, Data curation. **Puspa Lamsal:** Methodology, Formal analysis, Data curation. **Anita Bhattarai:** Methodology. **Aayush Bhattarai:** Writing – original draft, Methodology, Formal analysis, Data curation. **Shankar Tripathi:** Writing – review & editing, Writing – original draft, Methodology.

## Declaration of competing interest

The authors declare that this article's content has no conflicts of interest.
